# CD123-Directed Bispecific Antibodies for Targeting MDS Clones and Immunosuppressive Myeloid-Derived Suppressor Cells (MDSC) in High-Risk Adult MDS Patients

**DOI:** 10.3389/fragi.2021.757276

**Published:** 2021-09-27

**Authors:** Fatih M. Uckun, Justin Watts

**Affiliations:** ^1^ Aptevo Therapeutics, Seattle, WA, United States; ^2^ Immuno-Oncology Program, Ares Pharmaceuticals, St. Paul, MN, United States; ^3^ University of Miami Sylvester Comprehensive Cancer Center, Miami, FL, United States

**Keywords:** CD123, bispecific antibody, MDSC, MDS, T-cells, APVO436

## Abstract

There is an urgent need to identify effective strategies to prevent leukemic transformation and induce sustained deep remissions in adult high-risk myelodysplastic syndrome (MDS) patients. This article discusses the clinical impact potential of bispecific antibodies (BiAB) capable of redirecting host T-cell cytotoxicity in an MHC-independent manner to malignant clones as well as immunosuppressive myeloid-derived suppressor cells (MDSC) as a new class of anti-MDS drug candidates. T-cell engaging BiAB targeting the CD123 antigen may help delay disease progression in high-risk adult MDS and potentially reduce the risk of transformation to secondary AML.

## Introduction

Adult myelodysplastic syndrome (MDS), a heterogeneous group of clonal malignant hematologic disorders with an incidence rate of 4.5 per 100,000 persons per year, is characterized by ineffective hematopoiesis, abnormal differentiation of progenitor cells in the myeloid, erythroid, and megakaryocytic compartments, emergence of dysplastic myeloid cells, and an enhanced risk of transformation to acute myeloid leukemia (AML) (National Cancer Institute, 2016; National Cancer Institute, 2021; Zhan and Park, 2021). There is no effective standard treatment that will prevent the leukemic transformation or result in sustained deep remissions in high-risk adult MDS patients (Garcia-Manero, 2014; Götze and Platzbecker, 2018; Syed et al., 2020; Platzbecker et al., 2021). Several new therapies are being evaluated in clinical trials for their clinical impact potential for high-risk adult MDS patients, including new generation HMAs, isocitrate dehydrogenase inhibitors, the Hedgehog pathway inhibitor glasdegib, venetoclax plus azacitidine, CPX-351, the anti-CD47 monoclonal antibody magrolimab and the NEDD8 inhibitor pevonedistat, both in combination with azacitidine, and kinase inhibitors such as rigosertib, midostaurin, gilteritinib, and bemcentinib (Garcia-Manero, 2014; Götze and Platzbecker, 2018; Syed et al., 2020; Platzbecker et al., 2021; Sekeres et al., 2021).

**FIGURE 1:**
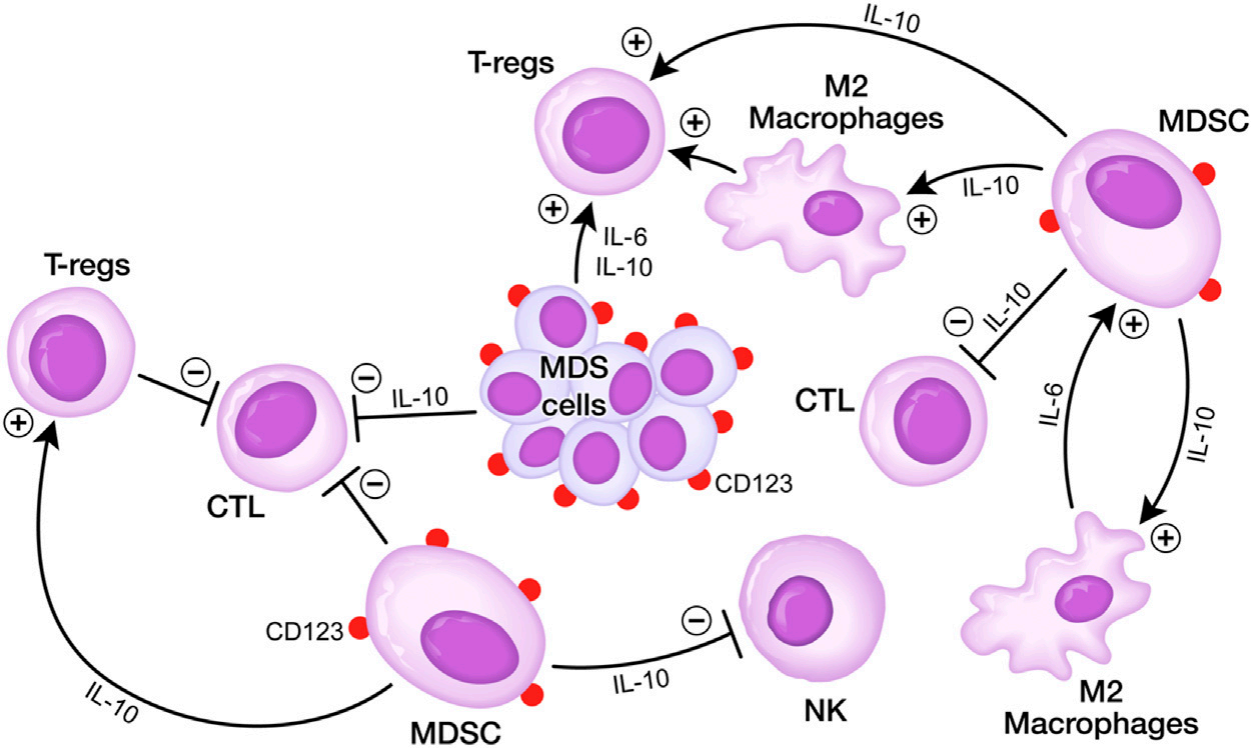
Immunosuppressive BMME in MDS. **(A)** MDS cells secrete several cytokines including IL-6 and IL10 that inhibit DCs, CTLs, but stimulate Tregs. MDSCs are stimulated via IL-6 by M2 macrophages and stimulate M2 macrophages as well as Tregs *via* IL10, but they inhibit *via* IL10 CTLs and NK cells. MDSCs express CD123 antigen on their surface which can be targeted by T-cell redirecting bispecific antibodies. See text for detailed discussion.

The immunosuppressive bone marrow microenvironment (BMME) in adult MDS has been implicated in clonal evolution and disease progression (Sallman and List, 2019; Younos et al., 2015; Chen et al., 2013; Gañán-Gómez et al., 2015). Expanded populations of myeloid-derived suppressor cells (MDSC), representing CD33^+^CD123^+^ immature myeloid cells within the bone marrow mononuclear cell fraction contribute to the immunosuppressive tumor microenvironment (TME) by inhibiting both memory and cytotoxic effector T-cell populations as well as natural killer (NK) cells, thereby promoting the immune evasion of MDS clones (Sallman and List, 2019; Younos et al., 2015; Chen et al., 2013; Gañán-Gómez et al., 2015; Parker et al., 2015; Movahedi et al., 2008; Marvel and Gabrilovich, 2015) (Figure 1). The abundance of MDSC is associated with a higher risk of rapidly progressive disease and poor survival outcomes in adult MDS (Chen et al., 2013; Younos et al., 2015; Sallman and List, 2019).

The α-chain of the IL-3 receptor, also known as the CD123 antigen, is broadly expressed on the bulk population of malignant MDS clones as well as leukemic blast and malignant hematopoietic stem and progenitor cells from AML patients (Jordan et al., 2000; Testa et al., 2002; Jin et al., 2009; Vergez et al., 2011; Hwang et al., 2012; Li et al., 2014; Testa et al., 2014; Chen et al., 2019). Furthermore, matched-pair analysis of samples from adult MDS patients who developed secondary AML with multi-parameter fluorescence-activated cell sorting and functional assays demonstrated that CD123 antigen is present on pre-malignant and malignant stem cells and blast cells in adult MDS (Chen et al., 2019). The expression of CD123 on MDSC as well as MDS clones provides a compelling rationale for targeting CD123 antigen on the malignant clones as well as the MDSC in the immunosuppressive BMME of adult MDS patients in an effort to delay disease progression and transformation to AML. Several biotherapeutic agents targeting CD123 have been developed and clinically evaluated in patients with AML and MDS, including the CD123-directed recombinant human IL3 fusion toxin Tagraxofusb (SL-401), monoclonal antibodies, bispecific antibodies targeting CD123 antigen, such as bispecific T-cell engagers, dual affinity retargeting antibodies, bispecific killer cell engagers, and tri-specific killer cell engagers (AL Hussaini et al., 2013; Kovtun et al., 2018; Comeau et al., 2019; Aldoss et al., 2020; Bai et al., 2020; Uy et al., 2021a; Daver et al., 2021; Isidori et al., 2021; Tabata et al., 2021). Antibody-drug conjugates (ADC) are linked to cytotoxic agents to directly lyse targeted MDS/AML blasts. Bispecific T-cell engagers (BiTE) and bi- and tri-specific NK cell engagers (BiKE, TriKE) bind and crosslink target antigens to T- and NK effector cells to mediate targeted cell destruction.

As shown in Table 1, a number of CD123-targeting biotherapeutics have entered clinical trials in adult MDS patients over the last 10 years. Flotetuzumab (MGD006) is a bispecific, dual-affinity retargeting (DART) antibody reactive with both CD3 antigen on T-cells and CD123 antigen on AML/MDS cells (Uy et al., 2021b). In a Phase 1 study in therapy-refractory adult AML and high-risk adult MDS patients (NCT02152956), this CD3-engaging bispecific antibody exhibited promising single agent activity with an overall composite response rate of 30% (Uy et al., 2017). Likewise, APVO436 showed promising single agent activity in high-risk MDS patients (Uckun et al., 2021). Of the 9 R/R AML/MDS patients treated at RP2D, 1 AML achieved a prolonged SD with time to progression of 238 days, two AML patients achieved a PR that deepened to a CR with full hematologic recovery, and one MDS patient achieved a marrow CR (Uckun et al., 2021). Among the six evaluable R/R MDS patients, three achieved a marrow CR, and the time to progression ranged from 78 to 321 days (Uckun et al., 2021). This dual-function MDS drug candidate is currently being evaluated in clinical trials for AML and MDS (NCT03647800). Vibecotamab (XmAb14045) is another CD123 × CD3 BiAB that has been evaluated clinically with a 23% CR rate in R/R AML patients (Testa et al., 2019). Other CD123 × CD3 BiAB in early-phase clinical trials in patients with R/R AML include SAR440334 (NCT03594955), a T-cell-engaging multispecific monoclonal antibody, and JNJ-63709178 (NCT02715011), a humanized DuoBody.

**TABLE 1:** Interventional CD123-Targeting biotherapy trials in MDS patients since 2011.

Protocol/Trial ID	Trial title	Trial phase	Primary tested drug	Trial objective	Start date	Supporting URLs
NCT04109482	A phase I/II, Open Label, Multicenter Trial to Assess the Safety and Efficacy of MB-102 in Patients With Relapsed or Refractory Blastic Plasmacytoid Dendritic Cell Neoplasm	I/II	MB-102, Mustang Bio	To assess the safety and efficacy of MB-102 in patients with relapsed or refractory BPDCN, AML or high-risk MDS.	February 17, 2020 (Actual)	https://clinicaltrials.gov/show/NCT04109482
			CD123 CAR-T			
NCT03594955	An Open-label, First-in-human, Dose Escalation Study of SAR440234 Administered as Single Agent by Intravenous Infusion in Patients With Relapsed or Refractory Acute Myeloid Leukemia (R/R AML), B-cell Acute Lymphoblastic Leukemia (B-ALL), or High Risk Myelodysplasia (HR-MDS)	I/II	SAR-440234	To determine the maximum tolerated dose (MTD) of SAR440234 administered as a single agent in patients with R/R AML (relapsed or refractory acute myeloid leukemia), HR-MDS (high risk myelodysplastic syndrome), or B-ALL (B-cell acute lymphoblastic leukemia), and determine the recommended phase 2 dose (RP2D) for the subsequent Expansion part. To assess the activity of single agent SAR440234 at the RP2D in patients with R/R AML or HR-MDS.	October 24, 2018 (Actual)	https://clinicaltrials.gov/ct2/show/study/NCT03594955
			CD3 × CD123 BiTE			
NCT03647800	Phase I/Ib Open-Label, Dose-Escalation Study of APVO436 in Patients With Relapsed or Refractory Acute Myeloid Leukemia (AML) or High-Grade Myelodysplastic Syndrome (MDS)	I	APVO-436	Part 1: To evaluate the safety and pharmacokinetic profile of APVO436 to determine a maximum-tolerated dose and recommended dose for part 2. Part 2: To assess the clinical activity and safety profile of APVO436 at the recommended dose in a larger group of patients.	December 13, 2018 (Actual)	https://clinicaltrials.gov/ct2/show/NCT03647800
			CD3 × 1CD123 BiAB			
NCT03113643	Phase 1 Study of SL-401 in Combination With Azacitidine or Azacitidine/Venetoclax in Relapsed/Refractory Acute Myeloid Leukemia (AML) or in Treatment-Naive Subjects With AML Not Eligible for Standard Induction Therapy or in Subjects With High-Risk Myelodysplastic Syndrome (MDS)	I/II	Tagraxofusp	To study SL-401 as a possible treatment for diagnosis of AML and high-risk MDS. To determine the safest, highest dose of study drug, SL-401, in combination with azacitidine that can be given to patients with AML or high-risk MDS. To study the side effects and best dose of DT(388)IL3 fusion protein SL-401 when given together with azacitidine in treating patients with myelodysplastic syndrome or acute myeloid leukemia that is untreated, has come back, or does not respond to treatment.	June 26, 2017 (Actual)	https://clinicaltrials.gov/show/NCT03113643
			DT388-IL3, SL-401, tagraxofusp-erzs			
NCT03011034	A phase II Proof-of-Concept Study to Separately Evaluate the Activity of Talacotuzumab (JNJ-56022473) or Daratumumab in Transfusion-Dependent Subjects With Low or Intermediate-1 Risk Myelodysplastic Syndromes (MDS) Who Are Relapsed or Refractory to Erythropoiesis-Stimulating Agent (ESA) Treatment	II	Daratumumab	To evaluate the efficacy (transfusion independence [TI]) of talacotuzumab (JNJ-56022473) or daratumumab in transfusion-dependent participants with low or intermediate-1 risk Myelodysplastic Syndrome (MDS) whose disease has relapsed during treatment with or is refractory to Erythropoiesis-Stimulating Agent (ESAs).	February 14, 2017 (Actual)	https://clinicaltrials.gov/show/NCT03011034
			Talacotuzumab			
			Anti-CD123 MoAb			
NCT02992860	Single Agent JNJ-56022473 in MDS and AML Patients Failing Hypomethylating Agent Based Therapy	II	Talacotuzumab	To evaluate the effect of JNJ-56022473 in overall hematological response rate at 3 months in HMA refractory/relapsed AML and MDS patients.	January 7, 2016 (Actual)	https://clinicaltrials.gov/show/NCT02992860
			Anti-CD123 MoAb			
NCT02181699	Phase I Study of KHK2823 in Patients With Acute Myeloid Leukemia or Myelodysplastic Syndrome	I	KHK-2823	To investigate the safety, pharmacokinetics, immunogenicity and pharmacodynamics of repeat doses of KHK2823.	January 6, 2014 (Actual)	https://clinicaltrials.gov/ct2/show/NCT02181699
			Anti-CD123 MoAb			
NCT02152956	A phase I/II, First in Human, Dose Escalation Study of MGD006, a CD123 × CD3 Dual Affinity Retargeting (DART^®^) Bi-Specific Antibody Based Molecule, in Patients With Relapsed or Refractory AML or Intermediate-2/High Risk MDS.	I/II	Flotetuzumab	To explore the ability of MGD006 to redirect T cells in relapsed and refractory acute myeloid leukemia. To see how the drug acts in the body (pharmacokinetics, pharmacodynamics) and to evaluate potential anti-tumor activity of Flotetuzumab.	September 6, 2014 (Actual)	https://clinicaltrials.gov/show/NCT02152956
			CD3 × CD123 DART			

Like other CD3 engaging BiAb, CD123 × CD3 BiAB are also associated with cytokine release syndrome (CRS) as a treatment-emergent and potentially life-threatening complication (Uy et al., 2017; Testa et al., 2019; Uy et al., 2021b; Uckun et al., 2021). CRS was observed in 96% of AML patients treated with Flotetuzumab (Uy et al., 2017) and 58% of AML patients treated with Vibecotamab (XmAb14045) (Testa et al., 2019). Within the confines of a small patient and heterogeneous patient population, the CRS rate of 21.7% in the Phase 1B study of APVO436 appeared to compare favorably with the reported CRS rates for the anti-AML bispecific antibodies (Uckun et al., 2021). Intriguingly, none of the 7 MDS patients treated with APVO436 experienced CRS (Uckun et al., 2021). APVO436-related CRS was not required for clinically meaningful responses in R/R AML patients, and it did not affect their survival outcome. Prolonged stabilization of disease, partial remissions and complete remissions were achieved in both patients who experienced CRS as well as patients who did not experience CRS after APVO436 infusions (Uckun et al., 2021). The predominant proinflammatory cytokine in CRS events associated with CD123xCD3 BiAB appears to be interleukin 6 (IL-6) and therefore tocilizumab at standard doses combined with dexamethasone. is commonly used to manage this potential complication (Testa et al., 2019; Uckun et al., 2021).

## Discussion

**FIGURE 2:**
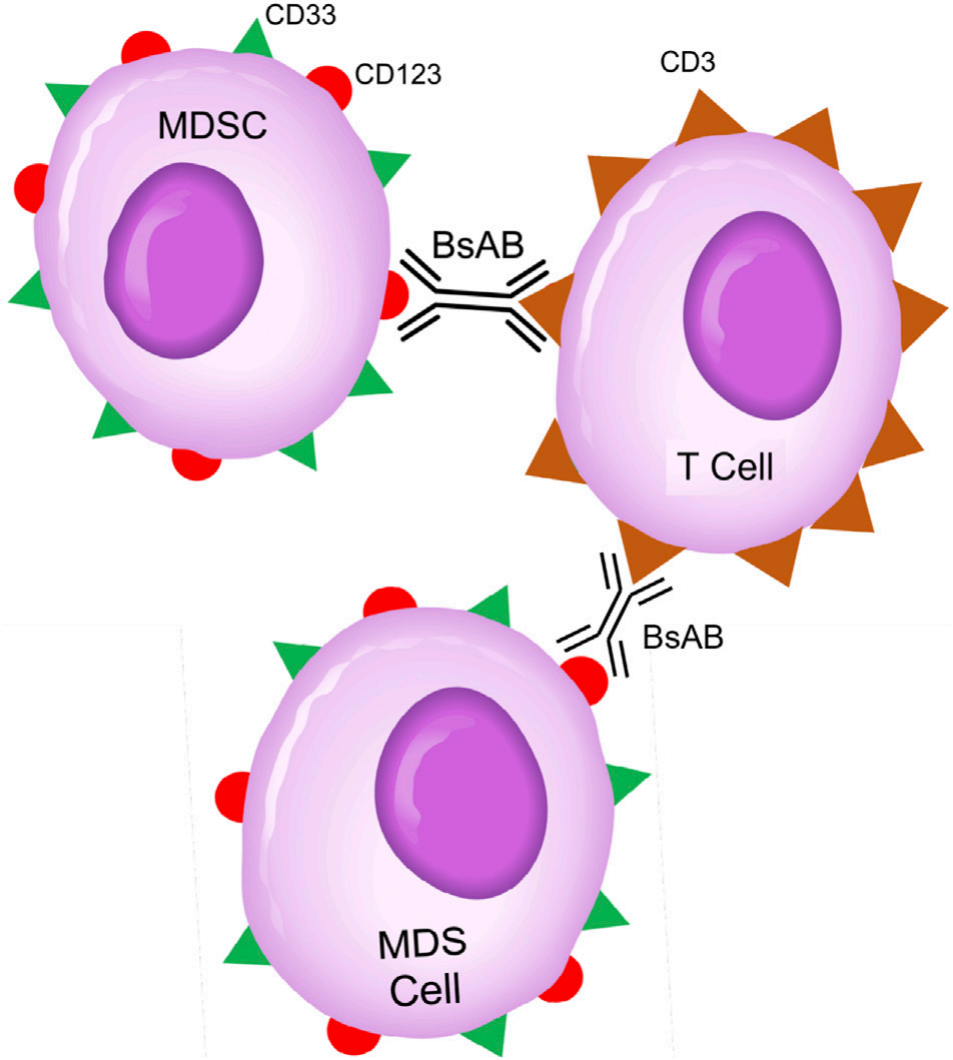
Bispecific CD3 × CD123 Antibodies Targeting MDS Clones and MDSC Cells in High-Risk MDS Patients. Abbreviations: BsAB: bispecific antibody; MDS: MDS clone; MDSC: Myeloid-derived suppressor cell. See text for a detailed discussion of the rationale of targeting the CD123 and CD33 antigens that are expressed on both MDS clones and MDSCs.

CD123-targeting, CD3-engaging BiAB bring cytotoxic T-cells (CTLs) within close vicinity of target CD123^+^ cells to create “cytolytic synapses” as a short bridge between MDS cells and CTLs, triggering CTL activation and destruction of targeted MDS cells (Figure 2). APVO436 is a recombinant T-cell engaging humanized BiAB designed to redirect host T-cell cytotoxicity in an MHC-independent manner to CD123-expressing blast cells from patients with hematologic malignancies (Comeau et al., 2019; Uy et al., 2021b; Uy et al., 2017; Uckun et al., 2021) (Figure 2). APVO436 was generally well tolerated in adults with relapsed AML and high-risk MDS with manageable toxicity and a promising benefit to risk profile (Uckun et al., 2021). APVO436 at the recommended Phase 2 dose (RP2D) level also produced early evidence of clinical efficacy. Both prolonged SD and CRs were observed as early evidence of clinical efficacy in R/R AML patients. Further, of the 6 MDS patients evaluable for response, three achieved a marrow CR (Uckun et al., 2021).

T-cells in the immunosuppressive BMME of AML and high-risk MDS patients with markedly deregulated innate and adaptive immune responses are characterized by a phenotype of “exhaustion” with augmented expression of PD-1, T cell immunoglobulin and ITIM domain (TIGIT), and T-cell immunoglobulin and mucin-domain containing-3 (TIM-3) antigens as well as functional cytotoxic T-cell (CTL) deficiency (Brauneck et al., 2021; Goswami et al., 2016; Ladikou et al., 2020; Jia et al., 2018). Furthermore, MDS patients treated with HMAs were reported to have increased PD-1 expression on peripheral blood mononuclear cells (Yang et al., 2014). The proof-of-concept that the CD3-engaging bispecific antibody APVO436 can sufficiently enhance the cytotoxicity of the dysfunctional and exhausted T-cells in relapsed AML patients and induce CRs in R/R AML patients and marrow CRs in MDS demonstrates its clinical impact potential for the treatment of CD123-expressing hematologic malignancies. The combination of APVO436 with immune-checkpoint inhibitors and/or CAR-T/CAR-NK cells may result in greater anti-leukemic activity, but the safety and tolerability of such combination immunotherapy awaits clinical confirmation.

We hypothesize that the addition of CD3 × CD123 BiABs will eradicate residual CD123^+^ MDS clones as well as leukemic stem cells. Notably, Venetoclax has recently been shown to augment T-cell effector function by increasing the production of reactive oxygen species and Azacitidine has been shown to enhance sensitivity of AML cells to cytotoxic T-cells by activating the STING pathway (Lee et al., 2021). These observations provide a compelling rationale for combining BiABs such as Flotetuzumab and APVO436 with Venetoclax, Azacitidine, or both to treat newly diagnosed high-risk MDS patients. Recently, Ganesan et al. reported a new and promising CD123 targeting BiAB platform aimed at selective recruitment of Vγ9^+^ γδ T cells as an alternative to CD3-engaging anti-CD123 BiABs which warrants clinical evaluation (Ganesan et al., 2021). In addition to CD123 targeting biotherapeutic agents, CD33 targeting antibodies and BiABs (CD3 × CD33 or CD16 × CD33) also show potential for the treatment of high-risk MDS patients because of the high level expression on both MDS clones and MDSC (Eksioglu et al., 2017); (Gleason et al., 2014). The early stage trials listed in Table 1 have not been designed to specifically address the potential of reducing the size of the MDSC population in the bone marrow microenvironment of the adult MDS patient population. We recommend that hypothesis-generating or hypothesis-testing biomarker studies become an integral part of the clinical studies in the future to fully assess the clinical potential of CD123-targeting BiAB.

CD123 expression has not been extensively studied in pediatric MDS, including juvenile myelomonocytic leukemia (JMML) (Niemeyer et al., 2019). Louka et al. recently reported that the CD38^+^ myeloid progenitor compartment in JMML patients, including both common myeloid progenitor cells and granulocyte monocyte progenitors express CD123 (Louka et al., 2021). Therefore, it is possible that targeting CD123 may also have clinical potential in treatment of some pediatric patients with CD123^+^ MDS.

## Conclusion

Recombinant T-cell engaging humanized bispecific antibodies can be designed to redirect host T-cell cytotoxicity in an MHC-independent manner and in a target-dependent manner, selectively, to CD123-expressing blast cells in patients with hematologic malignancies.

They have clinical impact potential in high-risk MDS as it may both prevent disease progression/immune evasion by reducing the numbers of CD123^+^ MDSC (Edwards et al., 2010; Gustafson et al., 2015; Uhel et al., 2019; Dysthe and Parihar, 2020; Khan et al., 2020; Domagala et al., 2021) and delay the development of secondary AML by T-cell mediated destruction of CD123^+^CD34^+^ blast cells.
